# TREM2 as a Potential Immune-Related Biomarker of Prognosis in Patients with Skin Cutaneous Melanoma Microenvironment

**DOI:** 10.1155/2023/8101837

**Published:** 2023-01-27

**Authors:** Xinlin Zhu, Zhaoxiang Zeng, Min Chen, Xianzhen Chen, Dongying Hu, Weiwei Jiang, Mingwei Du, Tianyang Chen, Tiancheng Chen, Wanqing Liao, Chao Zhang, Ying Qu, Weihua Pan

**Affiliations:** ^1^Department of Dermatology, Shanghai Key Laboratory of Medical Mycology, Changzheng Hospital, Naval Medical University, Shanghai, China; ^2^Department of Vascular Surgery, Changhai Hospital, Naval Medical University, Shanghai, China; ^3^Department of Dermatology, Jinan Central Hospital, Shandong University, Jinan, China; ^4^Department of Hematology, Huashan Hospital, Fudan University, Shanghai, China

## Abstract

**Background:**

The skin cutaneous melanoma (SKCM) is a devastating form of skin cancer triggered by genetic and environmental factors, and the incidence of SKCM has rapidly increased in recent years. Immune infiltration of the tumor microenvironment is positively associated with overall survival in many tumors. Triggering receptor expressed on myeloid cells 2 (TREM2) is a transmembrane receptor of the immunoglobulin superfamily and a crucial signaling hub for multiple pathological pathways that mediate immunity. Although numerous evidences suggest a crucial role for TREM2 in tumorigenesis of some tumors, no systematic SKCM analysis of TREM2 is available. *Mehods*. The relationship between TREM2 expression and diagnostic and prognostic value of SKCM patients via using The Cancer Genome Atlas (TCGA) data. The expression level of TREM2 and clinical characteristic correlation in SKCM patients were assessed by the Wilcoxon rank sum test. The cox regression methods, Kaplan-Meier (KM), and log-rank test were used to assess the impact of TREM2 expression on the overall survival (OS). Furthermore, the Gene Set Enrichment Analysis (GSEA) and TIMER were performed to evaluate the enrichment pathways and potential functions and quantify the immune cell infiltration level for TREM2 expression.

**Results:**

The TREM2 in SKCM sample expression levels was significantly higher than in normal tissues. Moreover, this expression level of TREM2 was also associated with the BMI of SKCM patients. KM overall survival analysis and OS curve displayed that a high-level TREM2 expression was significantly correlated with a better SKCM prognosis of patients as compared with a low level of TREM2 expression. The GSEA analysis also revealed that TREM2 was associated with immune functions, such as neutrophil activation.

**Conclusion:**

TREM2 played a crucial role in SKCM, which might be a prognostic biomarker and correlated with immune infifiltrates in SKCM patients.

## 1. Introduction

The SKCM is a devastating cause of cancer deaths worldwide, representing a heavy global health burden. There is approximately 3 percent of all skin cancers but 72 percent of skin cancer deaths related to SKCM [1, 2]. However, the potential mechanisms of SKCM carcinogenesis and development are still unclear. Although numerous diagnosis methods including immunohistochemistry, blood biomarkers assays, immunofluorescence, gene detecting, mypath melanoma (a popular molecular method that can efficiently distinguish benign from malignant), and some treatment strategies have greatly advanced recently, SKCM is one of the most common tumors in adolescent living in the United States though its 5-year mortality rate is still in a relatively low level [3–6]. Therefore, it is urgent to explore and study the carcinogenic mechanism and treatment strategies of SKCM. The improvements in molecular identification assays and targeted therapy have significantly drawn people's attention to the emerging notion of “precision medicine” [7]. As an important factor of “precision medicine,” the tumor microenvironment (TME) plays a crucial part in tumor genesis and cancer progression due to its complicated composition [8]. Some researchers have explored the correlation between the prognosis and the crucial immunological biomarkers [9, 10]. Some studies indicated that the heterogeneity in the immune responses and components of SKCM were significant, which plays a vital role in the SKCM prognosis of the patients [11]. Among TME, some key genes that drive carcinogenesis may be considered underlying therapeutic targets, and the immune-related cells including macrophages, neutrophils, and T cells which appear to contribute more to the immunotherapy of some tumor [12–14]. The identification of the patient's TME effects and biological characteristics in SKCM remains a challenging part, but it seems vital to dig into further details of the genetic layer for the dynamic transition illustration of TME and predict the prognosis in patients with SKCM.

The TREM2 is a new group member of the myeloid cell expression trigger receptor family discovered in 2001, mainly expressed in macrophages and monocytes [15]. The TREM2-mediated pathway plays a vital role in macrophage activation [16]. Similarly, the combination of TREM2 and its ligand not only promotes tumorigenesis, progression, and the high expression of CD163 in tumor-associated macrophages but also amplifies the inflammatory response in the local tumor microenvironment [17]. The TREM2 as a tumor driver is usually overexpressed in numerous carcinoma including gastric, hepatic, and ovarian carcinoma. The overexpression of TREM2 always promotes the invasion, proliferation, and migration of tumor cells [18–20]. Interestingly, experimental models of hepatic carcinoma in the context of liver fibrosis yielded the opposing effect, with TREM2 promoting fibrosis but inhibiting tumorigenesis [19]. However, it is important to note that the relationship between TREM2 and the tumorigenesis of SKCM has not been well studied to date. Numerous findings indicated that TREM2 only plays as an oncogene function, but especially in the SKCM diagnosis as well as patients prognosis prediction, it plays as a promising biomarker [21]. Therefore, detailed analysis at the genetic level may shed light on the underlying mechanism of tumorigenesis and progression in SKCM patients.

This study evaluated the TREM2 expression prognostic nuance in SKCM by bioinformatics analysis of clinical and immune-related features from TCGA. The results also suggested that the TREM2 gene is one of the potential biomarkers and indicators for diagnosis and prognosis prediction and therefore improves the diagnosis and prognosis of SKCM patients.

## 2. Materials and Methods

### 2.1. Data Acquisition and the Human Protein Atlas

The RNA-seq expression (level 3) profiles and SKCM corresponding clinical information for patient datasets were downloaded from TCGA dataset (https://portal.gdc.com). The Human Protein Atlas offered a broad amount of proteomic and transcriptome information of distinct human samples, which consists of cell, tissue, and pathology. The RNA-seq data were normalized in TPM (Transcripts per million reads) format and the level of difference beteween samples were expressed with log2 coversion. To retain clinical information and remove duplicate samples, the profiles of gene expression and clinical information were also downloaded. There were a total of 813 normal and 468 tumor tissues gathered for the present study. Furthermore, the TREM2 expression influence on the immune microenvironment was studied by dividing the tumor tissues into two main groups based on the TREM2 expression. All databases used in the present study are publicly open-access and available; therefore, there was no need for the approval of the ethical review board.

### 2.2. Functional Enrichment Analysis or GSEA

The GSEA is used to define the concordant differentiation along with statistical significance between the biological states (including the previously defined set). In the present study, the GSEA was utilized for the identification of all the correlated genes for the expression of the TREM2 gene. The statistical significance for survival differences was also examined among the higher TREM2 groups (reference gene set: C2.cp.v7.2.symbols.gmt). Furthermore, the adjusted *p* value of <5% was considered statistically significant. Similarly, the false discovery rate of <25% reflected the significantly enriched gene sets that were studied in the present study.

### 2.3. Immune Infiltration Analysis by TIMER

The comprehensive resource “TIMER” was performed for the systematic analysis of immune infiltrates across the cancer type's spectrum (https://cistrome.shinyapps.io/timer/). The TIMER is an open website that covers a total of 32 types of cancer and comprehends 10,897 TCGA database samples, directing to evaluate the immune inner infiltrate abundance. A series of TREM2 expressions and their correlation with the immune infiltrates abundance that comprises B cells and CD4+ T cells were analyzed in various cancer types. The tumor purity and TREM2 gene expression relationship were also displayed in the present study.

### 2.4. Statistical Analysis

The level of TREM2 gene expression in SKCM patients was assessed by box plot analysis. The TREM2 expression cut-off value was chosen by the median gene expression. The clinical feature association and SKCM patient's gene expression were statistically analyzed by the cox regression and Wilcoxon signed-rank test. Overall survival (OS) rate among the low and high groups of TREM2 gene expression was measured with Kaplan-Meier analysis by using the log-rank test. To assess the expression level of the TREM2 gene diagnostic value, with the area under the received operating characteristic (ROC) curve, the ROC curve was used as the diagnostic value. The univariate and multivariate cox analysis was performed to screen potential clinic prognostic factors. For the prediction of 1-, 3-, and 5-year OS, a nomogram was created by linking the TREM2 expression value with other clinical variables. All statistical analyses were performed using the software “SPSS” (version 21.0). For statistical significance, the *p* value < 0.05 was considered for the analysis.

## 3. Result

### 3.1. Patient's Baseline Characteristics

For this study, in total, 468 patients were acquired that had clinical features (detail mentioned in Table 1). Among these patients, a total of 179 (38.2%) were female and 289 (61.8%) were male. The participant's median age was 58 years (ranging from 48 to 71). Overall 41 (11.4%) of the patients were in stage I, while in stage II and stage III, there were 78 (21.6%) and 90 (24.9%) patients, respectively. Similarly, a total of 152 (42.1%) patients were marked with stage IV while 234, 74, 49, and 54 patients were in N0, N1, N2, and N3 stages, respectively. The LUAD was 25.3 months (ranged 0–227 months) for the median follow-up of the patients.

### 3.2. High TREM2 Expression in SKCM

**Figure 1:**
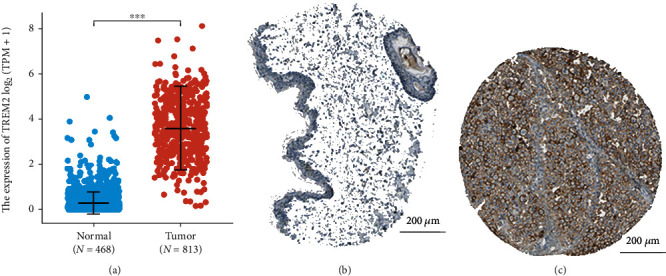
Analysis of the TREM2 expression in TCGA datasets and the Human Protein Atlas. (a) Validation of higher TREM2 mRNA expression in SKCM than that in normal tissue in TCGA datasets. (b, c) The level of TREM2 protein in melanoma tissue was higher than that in normal skin tissue in the Human Protein Atlas (antibody HPA010917).

**Figure 2:**
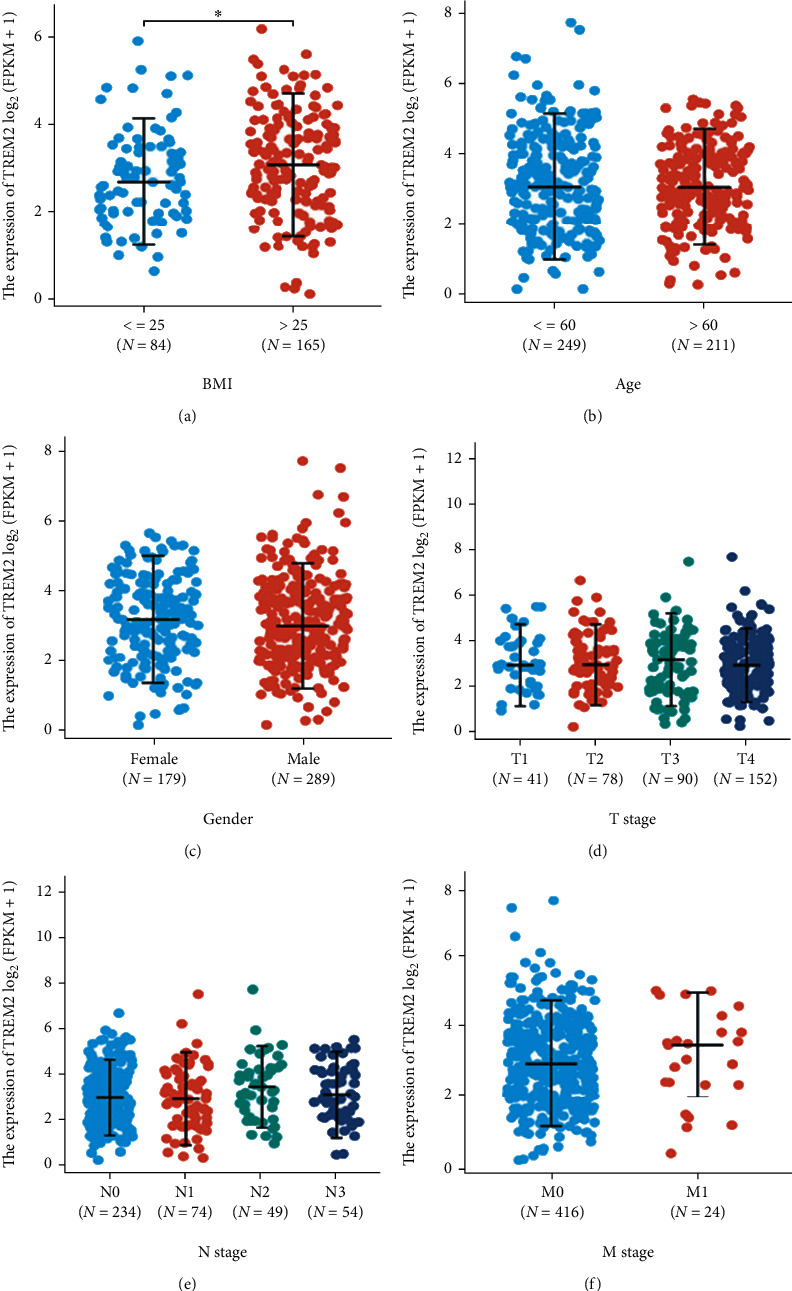
Association with TREM2 expression and clinicopathological characteristics. (a) There was a statistically significant difference between TREM2 mRNA levels and BMI. (b–f) There was no statistically significant difference between TREM2 mRNA levels and age, gender, and pathologic T, N, and M stage.

The status of SKCM patients with TREM2 expression was evaluated by comparing the expression level of TREM2 with the normal tissues. The analysis revealed that the level of TREM2 gene expression was statistically highly significant (*p* < 0.001) and greater in tissues of SKCM patients than in normal tissues (Figure 1(a)). Correspondingly, the expression of TREM2 protein is upregulated in Melanoma tissue in the Human Protein Atlas (Figures 1(b) and 1(c)). Furthermore, subgroup analysis in BMI, age, gender, pathologic T, N, and M stage. Similarly, significant differences were also observed between TREM2 mRNA levels and BMI (Figure 2), while there was no statistical difference between TREM2 mRNA levels and age, gender, and pathologic T, N, and M stage.

### 3.3. Correlation between Clinical Features and Expression Level of TREM2 Gene

From TCGA database, a total of 468 SKCM patients with both clinical and gene expression data were acquired (Table 2). The SKCM patients were equally in two groups (*n* = 234) “high-expression group” and the “low-expression group” based on the mean value of relative expression of TREM2. The correlation between the clinicopathological characteristics and TREM2 expression level of patients was also observed. The analysis showed that the expression level of TREM2 was not statistically affected (*p* > 0.05) by age, gender, race, BMI, and pathologic T, N, or M stage.

### 3.4. Low TREM2 Expression Is an Independent OS Risk Factor

**Figure 3:**
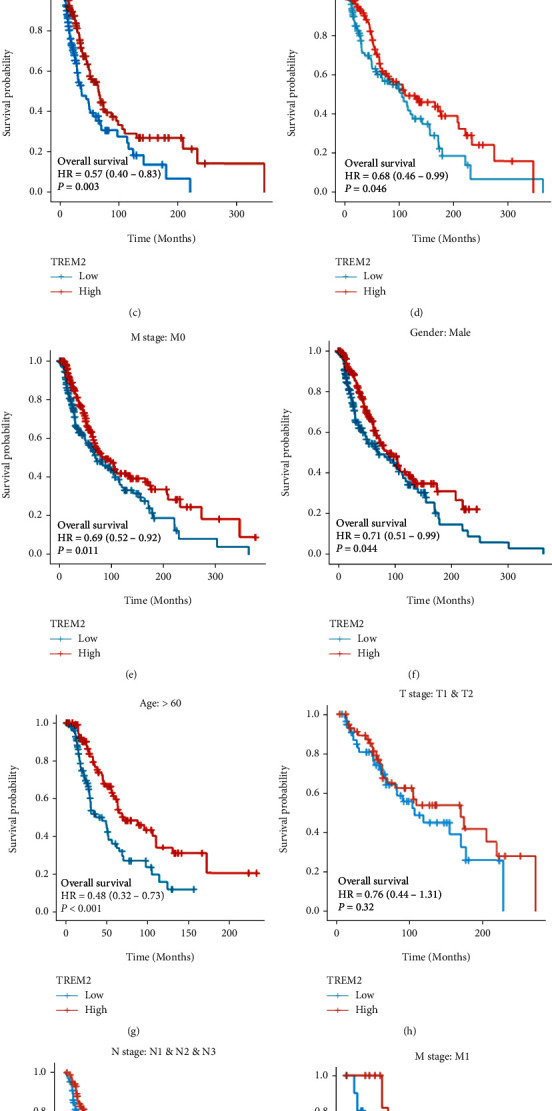
Kaplan-Meier curve for overall survival in SKCM. (a) Kaplan-Meier curve for TREM2 in all tumor patients. (b–j) Subgroup analysis for BMI ≤ 25, T3/T4, N0, M0, male, age greater than 60 years, T1/T2, N1/N2/N3, and M1.

The survival analysis by the Kaplan-Meier method also revealed that low expression of RRM2 was significantly correlated with the patient's poor prognosis (HR = 0.73, *p* = 0.024, Figure 3(a)). Furthermore, the clinical feature subgroup analysis also revealed that a high expression level of TREM2 was associated significantly with better SKCM prognosis in cases with BMI ≤ 25, T3/T4, N0, M0, M1, and male age greater than 60 years, as shown in Figures 3(b)–3(g) and 3(j). Moreover, the TREM2 expression has unaffected progress in the subgroup of T1/T2 and N1/N2/N3 (Figures 3(h) and 3(i)).

### 3.5. TREM2 Expression Diagnostic and Prognostic Value in SKCM

**Figure 4:**
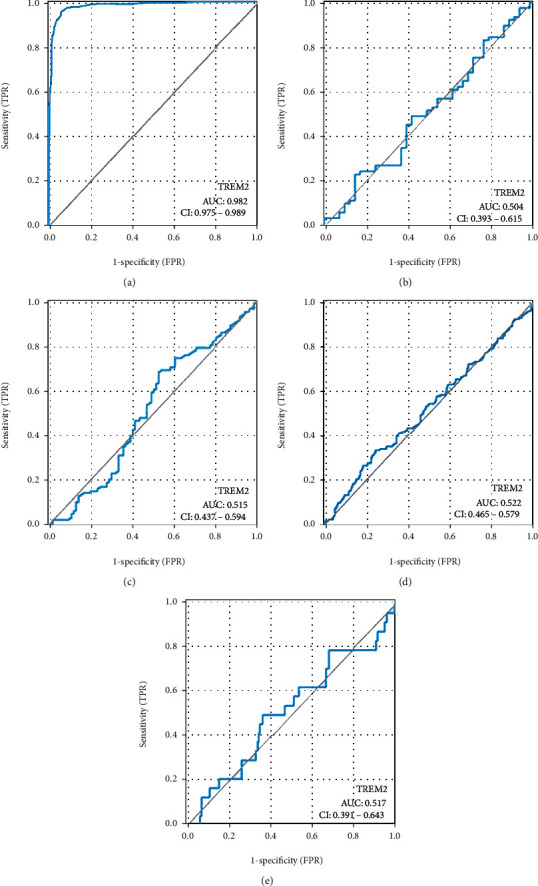
Diagnostic value of TREM2 expression in SKCM. (a) ROC curve for TREM2 in normal tissue and SKCM. (b–e) Subgroup analysis for T1/T2, T3/T4, N0/N1-N3, and M0/M1.

The TREM2 gene expression ROC curve analysis was used for the evaluation of the gene diagnostic value. The ROC curve (Figure 4(a)) area exhibited a high diagnostic value (0.982). The TREM2 expression diagnostic value in various SKCM clinical features, with AUC values of 0.504 for T1/T2, 0.514 for T3/T4, 0.522 for N0/N1-N3, and 0.517 for M0/M1 (Figures 4(b)–4(e)), was demonstrated by the subgroup analysis.

### 3.6. SKCM Prognostic Factor Analysis by Cox Univariate and Multivariate Method

**Figure 5:**
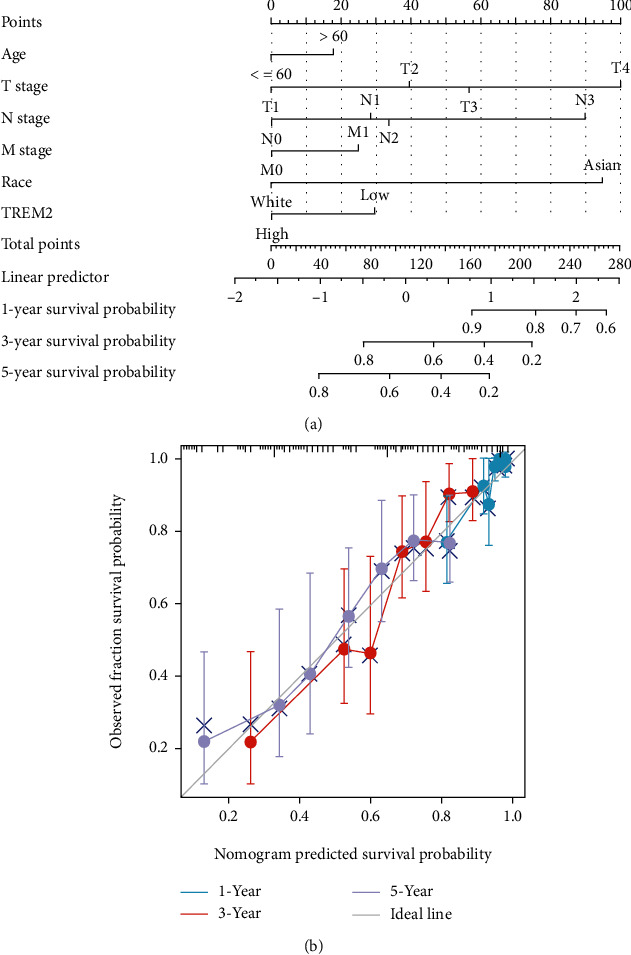
Nomogram for predicting the probability of patients with 1-, 3-, and 5-year overall survival. A quantitative method to predict SKCM patients' probability of 1-, 3-, and 5-year OS. (a) A nomogram for predicting the probability of 1-, 3-, and 5-year OS for SKCM patients. (b) Calibration plots of the nomogram for predicting the probability of OS at 1, 3, and 5 years.

The Cox univariate regression model with the variables age, extremities, T stage, N stage, M stage, pathologic stage, race, and TREM2 expression revealed high significance (*p* < 0.05, Table 3). Similarly, the multivariate Cox analysis further revealed that the expression in T, N, M, and pathologic stage, along with race and TREM2 in SKCM patients was independent OS prognostic factors. Hence, a nomogram was constructed for patients' prediction of 1-, 3-, and 5-year OS probability by combining the expression level of TREM2 with various clinical parameters (Figure 5(a)). The probability of survival in SKCM patients at 1, 3, and 5 years was determined by a vertical line directly down from the total point axis to the outcome axis. We also utilized the calibration curve to confirm the consistency between the actual survival probability and the predicted probability. And the result illuminated that the *C*-index of the model was 0.722 which suggested that the model is moderately accurate.

### 3.7. Identification of Signaling Pathway Related to TREM2

**Figure 6:**
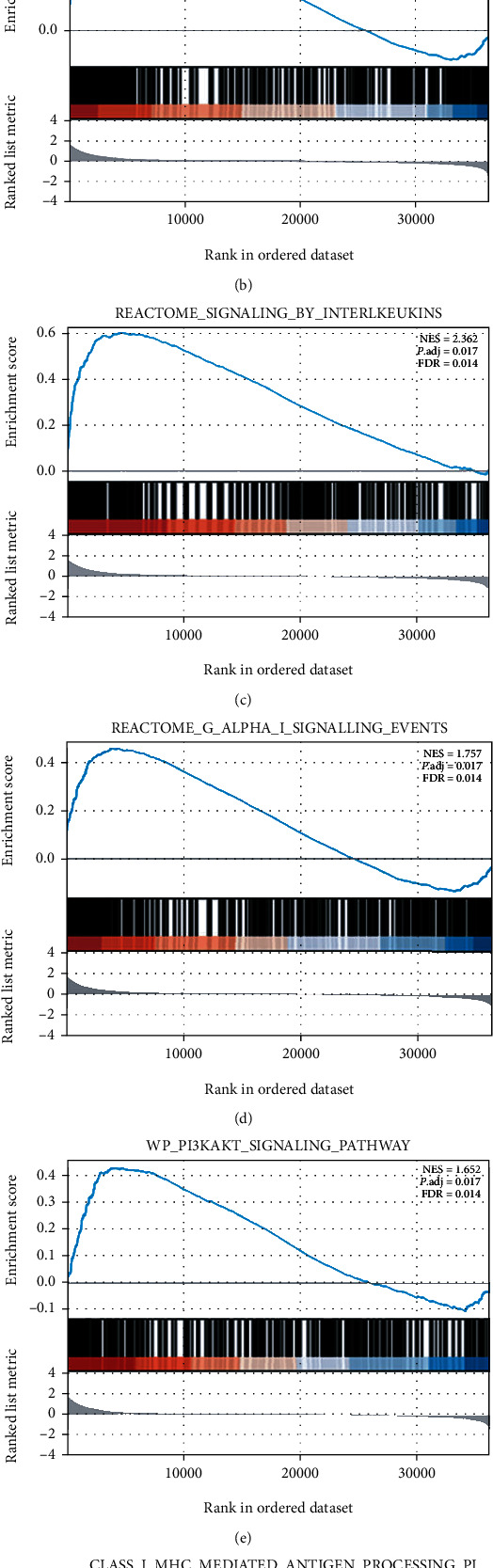
Enrichment plots from GSEA in the high TREM2 expression phenotype.

The data of high-TREM2 expression was evaluated by GSEA for the identification of signaling pathways that were differentially activated in SKCM patients. The GSEA revealed a high differentiation with false discovery rate less than 5% and adjust *p* value less than 25% in MSigDB Collections (c2.cp.v7.2.symbols.gmt). Furthermore, the gene sets related to the top 5 high TREM2 expression phenotypes are presented in Figure 6. There were “REACTOME_NEUTROPHIL_DEGRANULATION,” “REACTOME_GPCR_LIGAND_BINDING,” “REACTOME_SIGNALING_BY_INTERLEUKINS,” “REACTOME_G_ALPHA_I_SIGNALLING_EVENTS,” “WP_PI3KAKT_SIGNALING_PATHWAY,” “REACTOME_CLASS_I_MHC_MEDIATED_ANTIGEN_PROCESSING_PRESENTATION.” The results are summarized in Figure 6.

### 3.8. TREM2 Expression Is Correlated with Immune Infiltration Level and Cumulative Survival in SKCM

**Figure 7:**
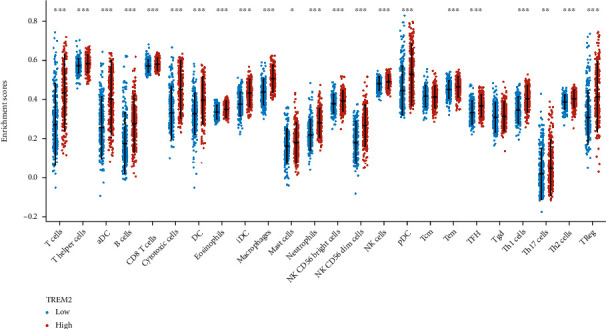
The proportion of 24 subpopulations of immune cells. Among them, Tcm and Tgd cells are similar in the high-expression group compared with the low-expression group (*p* > 0.05). In contrast, the rest 22 subpopulations of immune cells are a significant increase in the high-expression group compared with the low-expression group. ^∗^*p* < 0.05,  ^∗∗^*p* < 0.01, and^∗∗∗^*p* < 0.001.

To further detect the correlations between TREM2 expression and immune infiltration, ssGSEA analysis was conducted. Furthermore, for exploration of the gene expression profiles for the downloaded samples to infer the density of 24 types of immune cells the R package (RSVA) was utilized. Among them, Tcm (central memory T cells) and Tgd (dominant type of immunocytes residing in the intestinal epithelium) cells are similar in the high-expression group compared with the low-expression group (*p* > 0.05). In contrast, the rest 22 subpopulations of immune cells were significantly increased in the high-expression group compared with the low-expression group (Figure 7).

**Figure 8:**
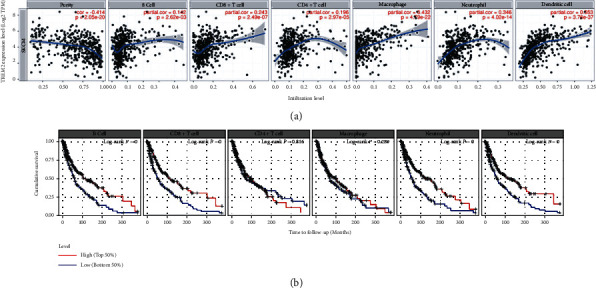
A TREM2 expression level has significant positive correlations with infiltrating levels of B cell, CD4+ T cells, macrophages, neutrophils, and DCs in SKCM. (a) Positive correlation exists between the TREM2 expression level and infiltrating levels of B cell (*r* = 0.142, *p* < 0.001), CD8+ T cells (*r* = 0.243, *p* < 0.001), CD4+ T cells (*r* = 0.196, *p* < 0.001), macrophages (*r* = 0.432, *p* < 0.001), neutrophils (*r* = 0.346, *p* < 0.001), and DCs (*r* = 0.553, *p* < 0.001). (b) Cumulative survival is related to B cell, CD8+ T cells, neutrophils, and DCs in SKCM.

The tumor-infiltrating lymphocytes independently predicted the sentinel lymph node status and OS among the patients. Further investigation was also done by TIMER for various cancer types, to evaluate the correlation between the immune infiltration levels and TREM2 gene expression. The TREM2 gene expression levels were selected that were positively correlated with tumor purity in various cancer types. The results also revealed that the TREM2 expression level was also correlated with high immune infiltration and better prognosis for patients with SKCM. Furthermore, a positive correlation also existed between the infiltrating levels of B cells (*r* = 0.142, *p* < 0.001), CD8+ T cells (*r* = 0.243, *p* < 0.001), CD4+ T cells (*r* = 0.196, *p* < 0.001), macrophages (*r* = 0.432, *p* < 0.001), neutrophils (*r* = 0.346, *p* < 0.001), and DCs (*r* = 0.553, *p* < 0.001) with the expression level of TREM2 in SKCM (Figure 8(a)). Additionally, the findings underline the DCs, CD8+ T cells, B cell, and neutrophil factors which were associated with the SKCM cumulative survival rate over time (Figure 8(b)).

## 4. Discussion

TREM2 is a member of the myeloid cell expression trigger receptor family, expressed primarily in macrophages and monocytes [15]. Meanwhile, TREM2 plays an important role in macrophage activation [16]. Rasmussen and Etzerodt [17] found that the combination between TREM2 and its ligand could promote tumorigenesis, progression, and the high expression of tumor-associated macrophages, implying that TREM2 was a key molecule in the progression of the tumor. For instance, various research studies have demonstrated that TREM2 is not only associated with inflammation but also promotes the proliferation, invasion, and migration of cells in numerous tumors [22, 23]. For example, Wang et al. [24] found that the TREM2 depletion can suppress the growth and invasion of glioma cells by Cromer invasion, apoptosis, and the KEGG chemokine pathway. Although overexpression of TREM2 is correlated with poorer prognosis in multiple tumors, including gastric, hepatic, and ovarian carcinoma, TREM2 has been shown to suppress tumorigenesis of hepatic carcinoma, in experimental models of hepatic carcinoma in the context of liver fibrosis [19]. Otherwise, Cheng et al. [21] found that high TREM2 had better OS in thyroid carcinoma and diffuse large B lymphoma through bioinformatics analysis. Hence, these studies suggest that TREM2 might have a protective effect on the prognosis of patients with tumors. Taken together, it has been proved that TREM2 might play a key role in the progression, tumorigenesis, and prognosis of numerous tumors.

The incidence of SKCM, one of the aggressive, therapy-resistant tumors of the human skin, has considerably increased during the past few decades [25, 26]. However, the prognosis of SKCM patients remains poor despite numerous advancements in diagnostic and treatment methods. Understanding the molecular mechanisms and the progression of the pathogenesis in SKCM and discerning promising biomarkers are crucial for exploring underlying therapeutic targets and improving the prognosis of SKCM patients. The genetics of SKCM involves genes related to pigmentation and naevi, as well as cell cycle genes that have been identified over the last decade [26]. Many gene pathways are being utilized for many new therapeutic targets, and boosting immune responses against the tumor seems to provide the best long-term effects [26]. Thus far, the expression of TREM2 and its underlying prognostic affecting on SKCM has not been extensively studied. Therefore, in the present investigation, we aimed to screen the TREM2 gene expression and underlying functions in SKCM, which may be conducted to the prognosis of SKCM patients, involving the overall survival and clinicopathological features. With the development of bioinformatics, numerous data can be obtained from public databases, herein, we performed a series of bioinformatics analyses using high-throughput RNA-seq data from TCGA. The database revealed that there were significant individual variations and heterogeneity in these data.

In this study, the level of TREM2 expression was found to be significantly higher in SKCM samples than in normal samples, and it was significantly associated with BMI. In addition, the association between the expression of TREM2 mRNA level and age, gender, and pathologic T, N, and M stage were not statistically significant (*p* > 0.05) which indicated that TREM2 expression was not affected by age, gender, race, BMI, pathologic T, N or M stage. Also, the AUC was 0.982, showing that the ROC curve for TREM2 performed well as a diagnostic tool. Interestingly, another key aspect of our study was that the expression of TREM2 was significantly associated with the overall survival of patients with SKCM and that the KM curve demonstrated longer overall survival for SKCM patients with higher TREM2 gene expression levels. Esparza-Baquer et al. [19] found that TREM2 plays a protective role in hepatocarcinogenesis via attenuated Wnt ligand secretion and inhibited hepatocyte proliferation and inflammation. Similar to the study of Esparza-Baquer et al. [19], it may mean that a high expression level of TREM2 could also inhibit tumorigenesis of SKCM and its upregulation was associated with considerably favorable survival in patients with SKCM. Consequently, our study revealed that TREM2 could serve as a promising moderate marker for the TME of SKCM and TREM2 might be a key predictor for prognosis in SKCM patients.

To study the potential functions and mechanisms of TREM2 in SKCM, we performed functional annotation based on GSEA for the exploration of the pathways enriched in high TREM2 expression samples. The analysis revealed that the high-level expression of TREM2 was associated with “immune functions” which include the “neutrophil degranulation” pathway and “signaling by interleukins” pathway, “GPCR ligand binding” pathway, “G alpha I signaling events” pathway, “PI3K/AKT” pathway, and “class I MHC (MHC-I) mediated antigen processing presentation” pathway. A series of studies have shown that neutrophil granule protein released after cell activation was significantly associated with tumor progression [27–29]. Besides, interleukins also played crucial roles in the progression of the tumor, such as interleukins can nurture an environment enabling and boosting tumor growth [30]. In addition, the PI3K/AKT pathway has been reported in various kinds of cancers [31, 32]. Similarly, the loss of functions of MHC-I antigen presentation in tumor cells can induce immunotherapy resistance and immune evasion [33]. Above all, these results demonstrated that TREM2 was involved in immune-related functions and significantly associated with tumors. Meanwhile, TREM2 may play a crucial role in SKCM by affecting these pathways, thus contributing to the prognosis of patients with SKCM.

Various studies have so far shown that immune infiltration can affect the prognosis of patients with tumors [34–36]. By employing TIMER, the analysis of the current study found that TREM2 expression was related to various immunological infiltration levels of macrophages, B cells, CD8+ T cells, CD4+ T cells, macrophages, neutrophils, and DCs. In addition, it was found that CD8+ T cells, neutrophils, B cells, and DCs were associated with the cumulative survival rate of SKCM patients, suggesting that high-level immune infiltration was advantageous for SKCM patients.

These analyses revealed that the TREM2 expression levels were significantly associated with tumor-infiltrating immune cells. It is possible for TREM2 to play a role in the immune response in SKCM, affecting the prognosis of SKCM patients. Altogether, these findings indicated that TREM2 might play a crucial role in the changes in the tumor immune microenvironment and the progression of SKCM. Overall, the expression of TREM2 may be a novel underlying factor for the immunotherapy of SKCM and may be a key component of a panel that reliably predicts the prognosis of SKCM patients.

Although these results improved our understanding of the association between TREM2 and SKCM, there were still some deficiencies. Firstly, relevant clinical factors, such as the details of treatment given to SKCM patients, should be considered to elaborate on the specific function of TREM2 in SKCM. Nevertheless, these data were lacking or incomplete in the database due to the patients being treated in different centers. In addition, the number of normal samples used as controls was quite different from that of SKCM samples; therefore, more studies are needed to maintain the balance of the samples. Finally, since only RNA-seq data from the database were used in this study, more research into the direct mechanism of TREM2 in SKCM will be required in the future.

## 5. Conclusion

The overexpression of TREM2 in SKCM may be a unique potential indicator for diagnosis, clinical prognosis, and therapeutic target of SKCM patients. However, further study should focus on the mechanism of TREM2 overexpression in SKCM. TREM2 also needs to be explored in the basic experiment to clarify the underlying mechanism of SKCM. This study provided a novel insight for further understanding the molecular pathogenesis of SKCM.

## Figures and Tables

**Table 1:** SKCM patient's clinical characteristics in the present study.

Characteristic	Levels	Overall
*n*		468

Age, *n* (%)	≤60	249 (54.1%)
	>60	211 (45.9%)

Gender, *n* (%)	Female	179 (38.2%)
	Male	289 (61.8%)

Race, *n* (%)	Asian	12 (2.6%)
	Black or African American	1 (0.2%)
	White	445 (97.2%)

BMI, *n* (%)	≤25	84 (33.7%)
	>25	165 (66.3%)

T stage, *n* (%)	T1	41 (11.4%)
	T2	78 (21.6%)
	T3	90 (24.9%)
	T4	152 (42.1%)

N stage, *n* (%)	N0	234 (56.9%)
	N1	74 (18%)
	N2	49 (11.9%)
	N3	54 (13.1%)

M stage, *n* (%)	M0	416 (94.5%)
	M1	24 (5.5%)

Pathologic stage, *n* (%)	Stage I	76 (18.6%)
	Stage II	140 (34.2%)
	Stage III	170 (41.6%)
	Stage IV	23 (5.6%)

Radiation therapy, *n* (%)	No	381 (82.6%)
	Yes	80 (17.4%)

Age, median (IQR)		58 (48, 71)

**Table 2:** The TREM2 expression and clinicopathological variables association in groups.

Characteristic	Low TREM2 expression	High TREM2 expression	*p* value
*n*	234	234	
Age, *n* (%)			1.000
≤60	124 (27%)	125 (27.2%)	
>60	106 (23%)	105 (22.8%)	
Gender, *n* (%)			0.342
Female	84 (17.9%)	95 (20.3%)	
Male	150 (32.1%)	139 (29.7%)	
Race, *n* (%)			0.771
Asian	7 (1.5%)	5 (1.1%)	
Black or African American	0 (0%)	1 (0.2%)	
White	222 (48.5%)	223 (48.7%)	
BMI, *n* (%)			0.050
≤25	53 (21.3%)	31 (12.4%)	
>25	81 (32.5%)	84 (33.7%)	
T stage, *n* (%)			0.629
T1	24 (6.6%)	17 (4.7%)	
T2	40 (11.1%)	38 (10.5%)	
T3	42 (11.6%)	48 (13.3%)	
T4	80 (22.2%)	72 (19.9%)	
N stage, *n* (%)			0.297
N0	119 (29%)	115 (28%)	
N1	39 (9.5%)	35 (8.5%)	
N2	18 (4.4%)	31 (7.5%)	
N3	26 (6.3%)	28 (6.8%)	
M stage, *n* (%)			0.852
M0	208 (47.3%)	208 (47.3%)	
M1	11 (2.5%)	13 (3%)	

**Table 3:** OS and clinical characteristic association by Cox regression analyses.

Characteristics	Univariate analysis		Multivariate analysis	
	Hazard ratio (95%, CI)	*p* value	Hazard ratio (95%, CI)	*p* value
Gender/sex				
Female	Reference			
Male	1.164 (0.872-1.554)	0.301		
Age				
≤60	Reference			
>60	1.678 (1.266-2.225)	<0.001	1.292 (0.915-1.825)	0.146
BMI				
≤25	Reference			
>25	0.819 (0.508-1.321)	0.414		
Tumor tissue site				
Extremities	Reference			
Trunk	0.943 (0.694-1.281)	0.707		
Head and neck	1.222 (0.718-2.082)	0.460		
Other specify	2.109 (1.095-4.062)	0.026		
T-stage				
T1	Reference			
T2	1.519 (0.824-2.800)	0.180	2.018 (1.014-4.017)	0.046
T3	2.069 (1.141-3.752)	0.017	3.391 (1.396-8.237)	0.007
T4	3.673 (2.047-6.591)	<0.001	6.640 (2.752-16.021)	<0.001
N stage				
N0	Reference			
N1	1.487 (1.007-2.197)	0.046	2.677 (0.907-7.906)	0.075
N2	1.525 (0.967-2.406)	0.069	2.786 (0.918-8.452)	0.070
N3	2.578 (1.651-4.028)	<0.001	6.361 (2.085-19.413)	0.001
M stage				
M0	Reference			
M1	1.734 (0.915-3.287)	0.092	0.608 (0.142-2.598)	0.502
Radiation therapy				
No	Reference			
Yes	0.953 (0.674-1.348)	0.785		
Pathologic stage				
Stage I	Reference			
Stage II	1.566 (1.041-2.356)	0.031	0.603 (0.288-1.261)	0.179
Stage III	1.943 (1.315-2.871)	<0.001	0.374 (0.112-1.246)	0.109
Stage IV	3.174 (1.567-6.428)	0.001		
Race				
Asian	Reference			
Black or African American	NA (NA-NA)			
White	0.223 (0.103-0.483)	<0.001	0.265 (0.101-0.695)	0.007
TREM2				
Low	Reference			
High	0.734 (0.561-0.961)	0.024	0.635 (0.459-0.879)	0.006

## Data Availability

No data were used to support this study.

## Abbreviations

TREM2: Triggering receptor expressed on myeloid cells 2
SKCM: Skin cutaneous melanoma
TCGA: The Cancer Genome Atlas
GSEA: Gene set enrichment analysis
OS: Overall survival
KM: Kaplan-Meier
ROC: Received operating characteristic
Tcm: Central memory T cells
Tgd: Dominant type of immunocytes residing in the intestinal epithelium.

## References

[1] Dzwierzynski W. W. (2013). Managing malignant melanoma. *Plastic and Reconstructive Surgery*.

[2] Huang B., Han W., Sheng Z. F., Shen G. L. (2020). Identification of immune-related biomarkers associated with tumorigenesis and prognosis in cutaneous melanoma patients. *Cancer Cell International*.

[3] Weinstein D., Leininger J., Hamby C., Safai B. (2014). Diagnostic and prognostic biomarkers in melanoma. *The Journal of Clinical and Aesthetic Dermatology*.

[4] Abbas O., Miller D. D., Bhawan J. (2014). Cutaneous malignant melanoma. *The American Journal of Dermatopathology*.

[5] Clarke L. E., Warf M. B., Flake D. D. (2015). Clinical validation of a gene expression signature that differentiates benign nevi from malignant melanoma. *Journal of Cutaneous Pathology*.

[6] Bleyer A., Viny A., Barr R. (2006). Cancer in 15- to 29-year-olds by primary site. *The Oncologist*.

[7] Moreira A. L., Eng J. (2014). Personalized therapy for lung cancer. *Chest*.

[8] Zhou M., Zhang Z., Zhao H., Bao S., Cheng L., Sun J. (2018). An immune-related six-lncRNA signature to improve prognosis prediction of glioblastoma multiforme. *Molecular Neurobiology*.

[9] Jacquelot N., Seillet C., Wang M. (2021). Blockade of the co-inhibitory molecule PD-1 unleashes ILC2-dependent antitumor immunity in melanoma. *Nature Immunology*.

[10] Nguyen A. H., Koenck C., Quirk S. K. (2015). Triggering receptor expressed on myeloid cells in cutaneous melanoma. *Clinical and Translational Science*.

[11] Yang R., Wang Z., Li J. (2021). The identification of the metabolism subtypes of skin cutaneous melanoma associated with the tumor microenvironment and the immunotherapy. *Frontiers in Cell and Development Biology*.

[12] Chen X. J., Wu S., Yan R. M. (2019). The role of the hypoxia-Nrp-1 axis in the activation of M2-like tumor- associated macrophages in the tumor microenvironment of cervical cancer. *Molecular Carcinogenesis*.

[13] Huang W., Chen J. J., Xing R., Zeng Y. C. (2021). Combination therapy: future directions of immunotherapy in small cell lung cancer. *Translational Oncology*.

[14] Sharabi A. B., Lim M., DeWeese T. L., Drake C. G. (2015). Radiation and checkpoint blockade immunotherapy: radiosensitisation and potential mechanisms of synergy. *The Lancet Oncology*.

[15] Bouchon A., Hernández-Munain C., Cella M., Colonna M. (2001). A DAP12-mediated pathway regulates expression of CC chemokine receptor 7 and maturation of human dendritic cells. *The Journal of Experimental Medicine*.

[16] Audrain M., Haure-Mirande J. V., Mleczko J. (2021). Reactive or transgenic increase in microglial TYROBP reveals a TREM2-independent TYROBP–APOE link in wild-type and Alzheimer's-related mice. *Alzheimer's & Dementia*.

[17] Rasmussen R. K., Etzerodt A. (2021). Therapeutic targeting of tumor-associated macrophages. *Advances in Pharmacology*.

[18] Li C., Hou X., Yuan S. (2021). High expression of TREM2 promotes EMT via the PI3K/AKT pathway in gastric cancer: bioinformatics analysis and experimental verification. *Journal of Cancer*.

[19] Esparza-Baquer A., Labiano I., Sharif O. (2021). TREM-2 defends the liver against hepatocellular carcinoma through multifactorial protective mechanisms. *Gut*.

[20] Binnewies M., Pollack J. L., Rudolph J. (2021). Targeting TREM2 on tumor-associated macrophages enhances immunotherapy. *Cell Reports*.

[21] Cheng X., Wang X., Nie K. (2021). Systematic pan-cancer analysis identifies TREM2 as an immunological and prognostic biomarker. *Frontiers in Immunology*.

[22] Awed A. A., Hasoon H. A., Meanji F. A., Duhmaj M. B., Alamri K. S. (2021). Irreducible inflamed inguinal hernia with infected gangrenous omentum after laparoscopic appendectomy: a case report. *Journal of Surgical Case Reports*.

[23] Cines D. B., van der Keyl H., Levinson A. I. (1986). In vitro binding of an IgE protein to human platelets. *Journal of Immunology*.

[24] Wang X. Q., Tao B. B., Li B. (2016). Overexpression of TREM2 enhances glioma cell proliferation and invasion: a therapeutic target in human glioma. *Oncotarget*.

[25] Markovic S. N., Erickson L. A., Rao R. D. (2007). Malignant melanoma in the 21st century, part 1: epidemiology, risk factors, screening, prevention, and diagnosis. *Mayo Clinic Proceedings*.

[26] Ribero S., Glass D., Bataille V. (2016). Genetic epidemiology of melanoma. *European Journal of Dermatology*.

[27] Mollinedo F. (2019). Neutrophil degranulation, plasticity, and cancer metastasis. *Trends in Immunology*.

[28] Rawat K., Syeda S., Shrivastava A. (2021). Neutrophil-derived granule cargoes: paving the way for tumor growth and progression. *Cancer Metastasis Reviews*.

[29] Yee P. P., Wei Y., Kim S. Y. (2020). Neutrophil-induced ferroptosis promotes tumor necrosis in glioblastoma progression. *Nature Communications*.

[30] Briukhovetska D., Dörr J., Endres S., Libby P., Dinarello C. A., Kobold S. (2021). Interleukins in cancer: from biology to therapy. *Nature Reviews. Cancer*.

[31] Tan A. C. (2020). Targeting the PI3K/Akt/mTOR pathway in non-small cell lung cancer (NSCLC). *Thorac Cancer.*.

[32] Guerrero-Zotano A., Mayer I. A., Arteaga C. L. (2016). PI3K/AKT/mTOR: role in breast cancer progression, drug resistance, and treatment. *Cancer Metastasis Reviews*.

[33] Burr M. L., Sparbier C. E., Chan K. L. (2019). An evolutionarily conserved function of polycomb silences the MHC class I antigen presentation pathway and enables immune evasion in cancer. *Cancer Cell*.

[34] Fridman W. H., Pagès F., Sautès-Fridman C., Galon J. (2012). The immune contexture in human tumours: impact on clinical outcome. *Nature Reviews. Cancer*.

[35] Ye L., Zhang T., Kang Z. (2019). Tumor-infiltrating immune cells act as a marker for prognosis in colorectal cancer. *Frontiers in Immunology*.

[36] Fan Y., Liu B., Chen F. (2021). Hepcidin upregulation in lung cancer: a potential therapeutic target associated with immune infiltration. *Frontiers in Immunology*.

